# Commentary on “Protecting Against Unintended Risk From Student Quality Improvement Projects in Health Care”

**DOI:** 10.63116/ahisp-26-008

**Published:** 2026-05-11

**Authors:** Valerie J. M. Watzlaf

**Affiliations:** Department of Health Information Management, School of Health and Rehabilitation Sciences, University of Pittsburgh

**Keywords:** quality improvement, privacy, security

## Abstract

In this commentary, the author examines opportunities for extended research and discussion on the article entitled “Protecting Against Unintended Risk From Student Quality Improvement Projects in Health Care,” including protection of employee data and other types of sensitive information that may be included in quality improvement projects.

## Introduction

In the article titled “Protecting Against Unintended Risk From Student Quality Improvement Projects in Health Care,” Bryant-Gawthrop provides a comprehensive theory and practice report that presents interesting perspectives and offers manageable recommendations for academic and health care organizations overseeing students conducting quality improvement (QI) projects within their own workplaces. This much-needed article addresses an important consideration for educational programs as the demand for experiential learning grows and data sharing becomes more common.

The distinction between QI projects and clinical research has been discussed in the literature in the past several years.^1–13^ However, this article’s focus on the student-employee dual role is unique and deserves attention. This dual role creates additional risks because protected health information (PHI) can inadvertently cross into areas where it could be breached. The student-employee role introduces complexities, as these individuals often have broader access to sensitive data through their employment roles.

## Clarifying Dual-Role Risk

Bryant-Gawthrop provides an in-depth discussion of patient data used in QI projects and emphasizes the need for mechanisms to protect information as it is transferred to other devices or shared across platforms. Although this focus on patient data protection is critically important, the author could have also addressed the protection of employee data and other types of sensitive information that may be included in QI projects. Risks may arise that are uncommon in traditional internal QI initiatives, such as:

- academic submission requirements that may involve data transfer;
- potential publication of findings; and
- unclear delineation of institutional authority.

Valuable practical examples gleaned from the literature are provided in the article, including models that academic institutions, primarily graduate nursing programs, can adopt to foster collaboration and partnership between Institutional Review Boards (IRBs) and academic programs in determining whether QI projects must undergo IRB review. The principles discussed are applicable across health care professions, and broader guidance for the student-employee dual role, regardless of discipline, would further enhance the article’s practical application.

Recommendations are provided on governance models needed for student-employees conducting QI projects at their health care organizations. The models encourage collaboration and cooperation when addressing ethical and regulatory standards beyond the IRB to reduce unforeseen risks and to avoid overregulation of student-employee QI projects, which are understandably focused on PHI under the Health Insurance Portability and Accountability Act. However, the landscape often extends into other areas, including the following:

- workforce performance metrics;
- financial or proprietary dashboards;
- operational workflow data; and
- algorithmic or other predictive outputs.

## The Heatmap and 5Circle Student Employee Venn for Quality Improvement Model

In the article, Figure 1 is a useful heatmap described by Bryant-Gawthrop that illustrates scenarios demonstrating the need for governance structures to address access to PHI and possible publication in relation to training, resource awareness, contracts/agreements, and PHI security. This practical map highlights the need to strengthen governance as project complexity increases. This visual tool serves as a valuable decision-making aid for administrators and faculty navigating these complex situations.

The author recommends the 5Circle Student Employee Venn for QI model as a governance structure for student-employees conducting QI projects. This model promotes shared responsibilities among all relevant parties (students, faculty advisors, and health care organization supervisors) to ensure patient safety and data protection. It also includes actions that may be needed at each stage of the project life cycle, while addressing data flow and analysis outcomes in detail. By distributing responsibility across multiple parties, the 5Circle Student Employee Venn for QI model creates a system of checks and balances that reduces the likelihood of oversights while maintaining efficiency in the project approval process. However, responsibility and/or authority need clarification, in areas of overlap, when disputes arise and where accountability is ensured if safeguards fail.

## Academic Submission Standards

The treatment of final academic submissions is important. Submitting deliverables to learning management systems or student-owned devices brings risk. The governance structure should address the allowability of PHI in academic submissions, the standards for de-identification, secure storage expectations for academic artifacts, and any policies related to publication.

## Additional Considerations

The author discusses a complex and unique topic in a productive manner, providing academic programs and health care organizations with tangible, useful, and thought-provoking recommendations for engaging student-employees in QI project development. However, the discussion should not end here. The recommendations can and should be adapted to address emerging issues surrounding this topic as health care technology, artificial intelligence, data security requirements, and governance models continue to evolve.

## Conclusions

This article makes a significant contribution by addressing the unique challenges faced by student-employees conducting QI projects and by providing practical solutions to manage potential risks while supporting valuable learning experiences. Effective governance of student-employee QI projects requires balance: avoiding overregulation that stifles innovation while ensuring safeguards commensurate with modern data complexity. As academic programs continue to integrate experiential QI into degree requirements, institutions must adopt transparent, scalable, and technology-aware oversight mechanisms.

CE Quiz
